# Retinal detachment in UGH Syndrome after cataract surgery

**DOI:** 10.22336/rjo.2021.78

**Published:** 2021

**Authors:** Francisco Manuel Hermoso-Fernández, Carmen Gonzalez-Gallardo, María Cruz-Rojo

**Affiliations:** * Department of Ophthalmology, San Cecilio University Hospital, Granada, Spain

**Keywords:** UGH syndrome, UGH syndrome, cataract surgery

## Abstract

**Purpose:** To report a case of retinal detachment (RD) in a UGH Syndrome after cataract surgery and to emphasize special aspects of the management, along with factors that must be considered.

**Methods:** We present the case of a 56-year-old man who underwent cataract surgery with a capsulorhexis leak, but the implantation of the lens in the sac did not hinder. 8 months after surgery, he presented several episodes of hypertensive uveitis that produced a progression in the excavation of the optic nerve head from 3/10 to 9/10 despite the treatment with ocular hypotensive drugs. He went to the emergency department due to sudden loss of vision during which a complete hemovitreous with a zone of transillumination and atrophy of the temporal sector of the iris were observed. Echo-B showed inferior retinal detachment. The apposition of the intraocular lens over the temporal region of the iris was observed in anterior segment OCT. Scleral band pars plana vitrectomy surgery was performed.

**Results:** Currently, the VA RE was 6/10 with controlled IOP without the need for treatment, the excavation was 9/10 and it preserved an island of inferior paracentral vision in the perimetry. Vitrectomy favored the posterior displacement of the lens, avoiding friction of the iris, thus eliminating outbreaks of hypertensive uveitis.

**Conclusions:** It is important to be aware of the mechanisms of uveitis-glaucoma-hyphema (UGH) syndrome. It is necessary to identify postoperative signs to make a diagnosis as soon as possible. It is time to consider that UGH syndrome can be caused by any type of pseudophakic lens.

## Introduction

Ellingson or UGH syndrome is associated with the triad of: uveitis, glaucoma, and hyphema, although it may appear incompletely. It rarely appears as a subacute or chronic complication of cataract surgery, showing in weeks or years after IOL implantation. This disorder may be due to the inadequate size of the lens that exerts friction on the iridocorneal angle and/ or the vascular structures of the uvea, producing chronic inflammation, bleeding, and a blockage of the trabecular meshwork by erythrocytes or cellular debris with increased IOP and the appearance of glaucoma. In chronic cases, the posterior pole can be affected with vitreous hemorrhages and cystoid macular edema [**1**].

## Case presentation

**Fig. 1:**
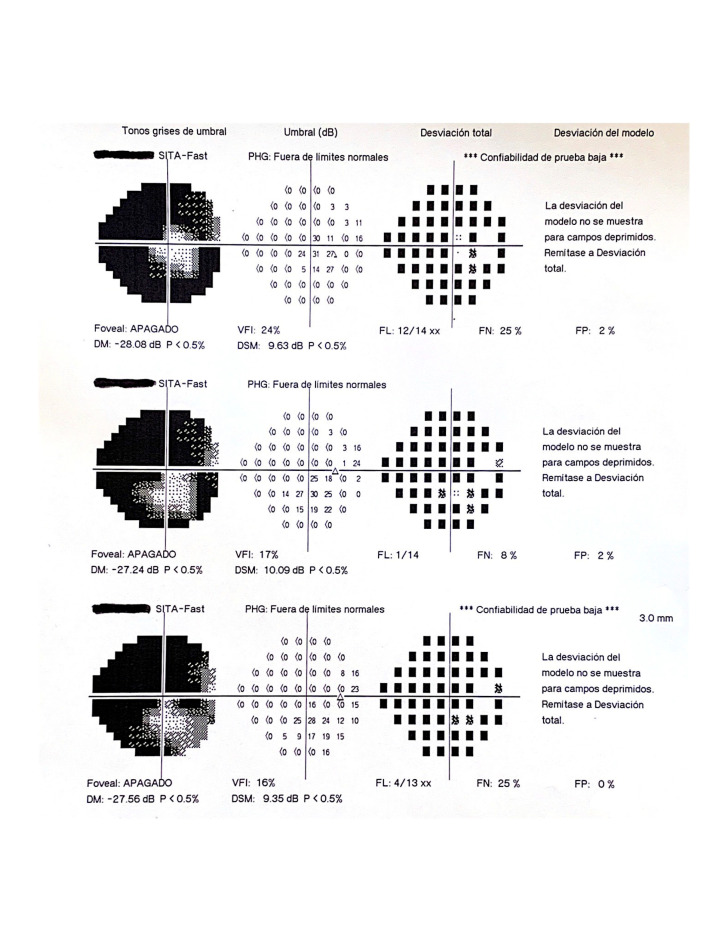
Visual field. Concentric reduction of the visual field with central 10º preservation

 A 56-year-old man who came to the emergency room with blurred vision, redness, and pain in his right eye for 3 days. As background, the patient had undergone cataract surgery in his RE 8 months before with anterior capsulorhexis leak as only complication. Despite that, the intraocular lens could be implanted in a sac. At the ophthalmological examination, the patient presented a VA of 6/5 in his RE. The biomicroscopy examination revealed: good anterior chamber depth, moderate posterior capsule opacity, ciliary hyperemia, Tyndall (++++) and grade II corneal edema. Posterior synechiae were not seen. His RE IOP was 34 mmHg. The fundus showed a papillary excavation of 0.3 papilla diameters, the rest of the posterior pole being normal. A possible case of acute anterior uveitis (AU) due to Posner Schlossmann syndrome was diagnosed and treatment was prescribed with dexamethasone, timolol and cyclopentolate eye drops. The patient had a good response to the prescribed treatment but with numerous recurrences, with high IOP peaks over a period of 9 years, during which he was evaluated on numerous occasions by the systemic diseases’ unit of our hospital, developing an axonal neuropathy with increased papillary excavation accompanied by a concentric reduction of the visual field with central 10º preservation, except for the upper nasal level, where complete quadrantanopia was observed (**Fig. 1**).

**Fig. 2:**
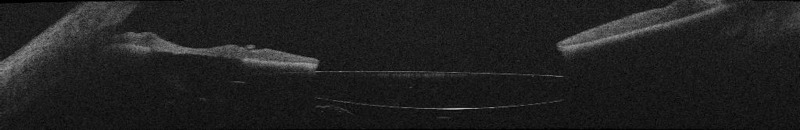
Temporal iris atrophy due to intraocular lens contact

The patient underwent a posterior capsulotomy with YAG laser in his RE and 10 days later he went to the emergency room of our hospital due to vision loss. The VA RE was 6/40. The biomicroscopy examination showed Tyndall (+++), abundant pigment in the endothelium, a zone of transillumination and atrophy of the temporal sector of the iris, in addition to the presence of vitreous at the lower pupillary level. A hemovitreous was observed in the fundus, that did not allow the correct visualization of the retina. Ultrasound B showed hemovitreous echoes and it was suggestive of retinal elevation at the lower level. A pars plana vitrectomy with a scleral band was considered. Intraoperatively, an inferior retinal detachment was found with the elevation of the inferior hemimacula to the foveal limit. The surgery was uneventful. Uveitis-Glaucoma-Hyphema (UGH) syndrome was then suspected given the evolution of the condition and the atrophic quadrant of the iris at the temporal level. When performing anterior segment OCT, we observed the atrophy area of the temporal sector of the iris due to contact with the intraocular lens (**Fig. 2**).

**Fig. 3:**
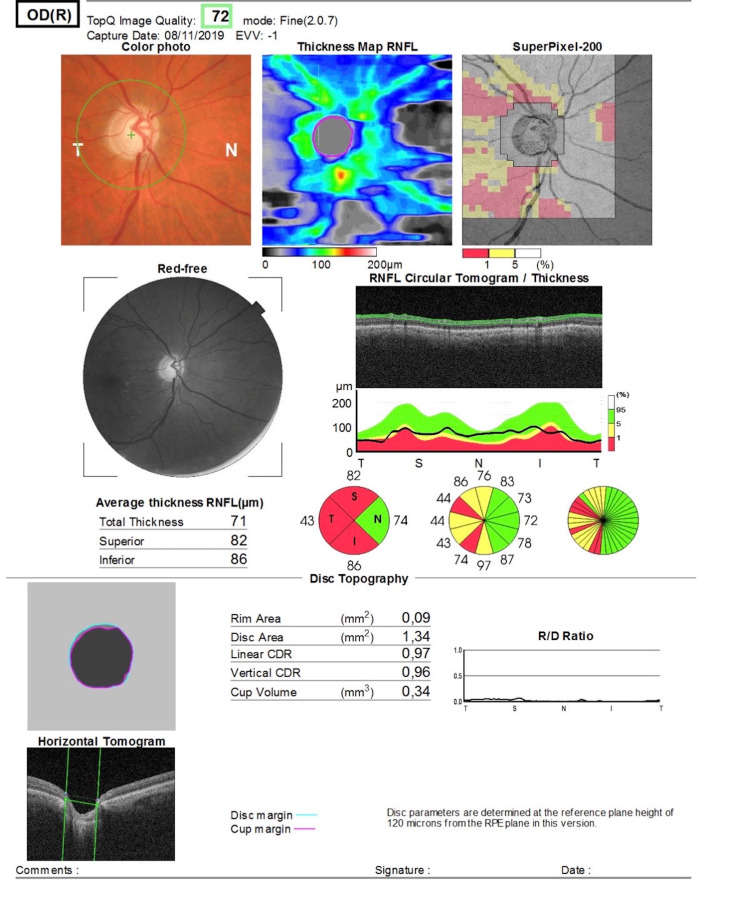
Optic Nerve OCD. The papilla shows an Inferior 67-micron, Superior 71-micron, Temporal 49-micron, and Nasal 57-micron disc thickening map

At that moment, the patient had a VA RE of 6/10, with good IOP control, the lens was kept in situ, and the visual field preserved an islet of inferior paracentral vision, the scotoma enlargement coinciding with the area affected by retinal detachment. The papilla showed an Inferior 67-micron, Superior 71-micron, Temporal 49-micron, and Nasal 57-micron disc thickness map (**Fig. 3**).

## Discussion

UGH syndrome is a rare complication of cataract surgery due to mechanical trauma to the intraocular lens. This type of complication is very rare and this turns it into a repeatedly underdiagnosed problem, as in the case that we presented. The consequences of this condition become disastrous in cases without early treatment, see this clinical case, which due to the tension peaks during uveitis outbreaks develop an axonal neuropathy with a marked reduction in the visual field, preserving only one lower paracentral islet of visual field. Therefore, it is vital to diagnose this syndrome early to avoid the serious consequences that it can produce. It is necessary to consider that UGH syndrome can be caused by any type of intraocular lens. In turn, in cases in which an intraocular lens is intracapsular and well positioned, it should not make us rule out the diagnosis of UGH. For this, it is vitally important to know the mechanisms of production of uveitis-glaucoma-hyphema syndrome (UGH), such as mechanical irritation caused by misposition of the IOL or subluxation that causes mechanical and repetitive trauma to the iris. Zonal laxity due to pseudo-exfoliation is caused by phacodonesis or previously rotated iris process, in the configuration of an iris plateau, and capsular fibrosis around the optics caused by contact at various points [**2**,**3**].

It is also essential to identify postoperative signs (transillumination, microhyphemas, pigment dispersion, iris neovascularization, macular cystoid edema, recurrent vitreous hemorrhage) to make a diagnosis as soon as possible. Before the surgery, it is necessary to know what factors can produce this pathology, as well as all those that make surgery difficult: zonular laxity, narrow pupil, anticoagulants, in addition to knowing the design and the lens that are implanted [**4**]. It is important to consider the use of alpha2 agonist treatments in these cases, to avoid the progression of axonal neuropathy attributed to ischemia generated by voltage spikes, since this has demonstrated neuroprotection of ganglion cells through direct inhibition of receptors NMDA in ischemic phenomena and glaucoma, as these are directly related to over-excitation of NMDA receptors [**5**]. On the other hand, in these cases the best treatment is surgical: by rotating the lens, or, if it is not enough, by removing the implant for replacement. In our case, it was not necessary at that moment because the vitrectomy produced a posterior displacement of the lens that led to the disappearance of outbreaks of hypertensive uveitis.

## Conclusion

It is necessary to be aware of the mechanisms of uveitis-glaucoma-hyphema (UGH) syndrome, such as: mechanical irritation caused by IOL malposition or subluxation provoking mechanical and repetitive trauma to the iris; IOL well positioned with proximity of the edge of the optic IOL edge to the lower pupillary margin, pressing on the peripheral iris; zonal laxity due to pseudoexfoliation caused by phacodonesis; previously rotated iris, plateau iris and fibrosis configuration capsule around the optics caused by contact at various points. It is essential to identify postoperative signs to make a diagnosis as soon as possible and to consider that UGH syndrome can be caused by any type of pseudophakic lens and an intraocular lens in the capsular bag should not rule out the diagnosis.

**Conflict of Interest statement**

None of the authors has any financial/ conflict of interest to disclose.

**Informed Consent and Human and Animal Rights statement**

Informed consent has been obtained from all individuals included in this study.

The patient offered her consent for the publication of her identifiable details in relation to the article “Retinal detachment in UGH Syndrome after cataract surgery” in Romanian Journal of Ophthalmology. 

He offered her consent for the publication of her identifiable details, including photograph(s) and/ or videos and/ or case history and/ or details in the text to be published in the above-mentioned journal. She discussed this consent form with Francisco Manuel Hermoso Fernández, the author of the paper.

**Authorization for the use of human subjects**

Ethical approval: The research related to human use complies with all the relevant national regulations, institutional policies, is in accordance with the tenets of the Helsinki Declaration, and has been approved by the review board of San Cecilio University Hospital, Granada, Spain.

**Acknowledgements**

None.

**Sources of Funding**

Authors have not received founding from any organization related (National Institutes of Health (NIH); Welcome Trust; Howard Hughes Medical Institute (HHMI).

**Disclosures**

The authors declare that they have no interest in relation to this article.
